# covidscreen: a web app and R Package for assessing asymptomatic COVID-19 testing strategies

**DOI:** 10.1186/s12889-022-13718-4

**Published:** 2022-07-15

**Authors:** Jesse Smith, Yilun Sun, Diego R. Hijano, James M. Hoffman, Hana Hakim, Richard J. Webby, Randall T Hayden, Aditya H. Gaur, Gregory T. Armstrong, Motomi Mori, Li Tang

**Affiliations:** 1 Department of Biostatistics, St. Jude Children’s Research Hospital, Mail Stop 768, 262 Danny Thomas Place, TN 38105 Memphis, USA; 2 Department of Infectious Diseases, St. Jude Children’s Research Hospital, Mail Stop 768, 262 Danny Thomas Place, TN 38105 Memphis, USA; 3 Department of Pharmacy and Pharmaceutical Sciences, St. Jude Children’s Research Hospital, Mail Stop 768, 262 Danny Thomas Place, TN 38105 Memphis, USA; 4 Department of the Office of Quality and Patient Safety, St. Jude Children’s Research Hospital, Mail Stop 768, 262 Danny Thomas Place, TN 38105 Memphis, USA; 5 Department of Pathology, St. Jude Children’s Research Hospital, Mail Stop 768, 262 Danny Thomas Place, TN 38105 Memphis, USA; 6 Department of Epidemiology and Cancer Control, St. Jude Children’s Research Hospital, Mail Stop 768, 262 Danny Thomas Place, TN 38105 Memphis, USA

**Keywords:** COVID-19, PCR test, Infection prevention, R, Shiny, Probabilistic model, Asymptomatic testing evaluation, Cost-effectiveness

## Abstract

**Background:**

COVID-19 has caused over 305 million infections and nearly 5.5 million deaths globally. With complete eradication unlikely, organizations will need to evaluate their risk and the benefits of mitigation strategies, including the effects of regular asymptomatic testing. We developed a web application and *R* package that provides estimates and visualizations to aid the assessment of organizational infection risk and testing benefits to facilitate decision-making, which combines internal and community information with malleable assumptions.

**Results:**

Our web application, covidscreen, presents estimated values of risk metrics in an intuitive graphical format. It shows the current expected number of active, primarily community-acquired infections among employees in an organization. It calculates and explains the absolute and relative risk reduction of an intervention, relative to the baseline scenario, and shows the value of testing vaccinated and unvaccinated employees. In addition, the web interface allows users to profile risk over a chosen range of input values. The performance and output are illustrated using simulations and a real-world example from the employee testing program of a pediatric oncology specialty hospital.

**Conclusions:**

As the COVID-19 pandemic continues to evolve, covidscreen can assist organizations in making informed decisions about whether to incorporate covid test based screening as part of their on-campus risk-mitigation strategy. The web application, *R* package, and source code are freely available online (see “Availability of data and materials”).

**Supplementary Information:**

The online version contains supplementary material available at 10.1186/s12889-022-13718-4.

## Background

As the COVID-19 pandemic’s third year begins, over 305 million known cases of the disease have been reported globally, and nearly 5.5 million of these people have died [1]. Estimates of excess deaths are even higher, at more than 19 million [2]. Massive efforts have made a COVID-19 vaccine available to over 4.5 billion people [3]. However, almost half of the global population has remained ineligible, unable or unwilling to be vaccinated, and the burden has been disproportionately observed in low-income countries [4]. In addition, the emergence of the Omicron variant of the virus has complicated recovery efforts by increasing the level of vaccination coverage needed, and future variants bring the possibility of further vaccine evasion, more severe disease, or other phenotypic changes [5].

Complete eradication of SARS-CoV-2 appears highly unlikely due to limited vaccination rate in certain regions or among the vulnerable population, waning immunity after infection, and continued emergence of new variants with phenotypic changes [4–7]. Instead, governments, societies, and organizations must adapt to the threat presented by COVID-19 while maintaining organizational operations and individual quality of life. Many organizations have required some combination of employee vaccination and regular testing; in the US, many organizations with federal contracts are mandated to do so [8]. An example organization requiring such measures are healthcare organizations, where there is often an increased risk of exposure, severe disease, or both. This testing strategy is commonly enhanced and supplemented by additional strategies, including sample pooling to increase testing capacity and smartphone-based screening tools to efficiently identify and isolate symptomatic individuals in time or to track symptom progression [9–12]. Such strategies work in tandem with asymptomatic testing but can also change the testing needs and testing capacity of an organization.

As a result, though many organizations previously or currently require regular asymptomatic COVID-19 testing among unvaccinated and some vaccinated employees, the value of such strategies has not been formally evaluated and remains unclear. At a minimum, this impairs decision-making through lack of information on true organizational risk and the benefits offered by asymptomatic testing. It is likely that some organizations will assertively judge an expensive testing strategy as too costly for the benefits; on the other hand, other organizations may adopt unnecessarily aggressive and costly testing strategies that far exceed their needs. With our web app, we aim to provide organizations with a flexible visual tool that can aid leaders in making decision throughout the pandemic.

### Covidscreen

A simulation-based approach was developed in 2020 to assess the benefits of different asymptomatic testing strategies in a hypothetical organization. The simulation used information on community case rates, asymptomatic-to-symptomatic case ratio, and assumed infectiousness periods to estimate the reduction in active cases on campus achieved by regular asymptomatic testing. It profiled the number of circulating active cases brought in from the community for a range of inputs, assuming the organization actively carries out other on-campus non-pharmaceutical intervention measures, such as mask mandate and timely testing as well as isolating symptomatic individuals, so that the on-campus transmission is reasonably assumed minimal and negligible. The results were intended to inform decisions on the type and strength of asymptomatic testing interventions needed.

With the future of COVID-19 uncertain, organizations may need to make such decisions repeatedly and in a variety of changing circumstances. To assist with these decisions, we present an application called covidscreen that combines information on a specific organizational setting with community context and assumption values which can be further customized by the user regarding the performance of various asymptomatic testing strategies. The application provides a graphical user interface (UI) that presents the estimated benefits of a given asymptomatic testing strategy, as well as untangling interactions between vaccination and testing benefits. It allows interested users with advanced knowledge to create risk profiles that illustrate relationships between risk, interventions, and projection assumptions.

### Similar work

Several applications have been developed to quantify individual-level risk of infection or exposure. One of the first and most well-known is a tool from Chande et al. that combines real-time data from aggregation sites with a binomial model to estimate the risk of exposure for a given event size in different areas of the United States [13]; a similar tool calculates the risk of infection and compares it with a “risk budget” [14]. Other tools estimate the risk of severe disease using detailed self-reported data [15, 16]. Paltiel et al. and others have addressed cost-effectiveness directly in the literature but do not provide tools to make such assessments accessible and customizable by decision-makers [17].

Few user-friendly tools have been developed to help quantify and visualize estimated organizational risk associated with a given context or the expected benefits of a selected asymptomatic testing strategy. covidscreen fills such a gap. Below, we give an overview of this functionality through a case study of asymptomatic employee testing program at St. Jude Children’s Research Hospital, a pediatric specialty hospital located in Memphis, Tennessee, USA. Following, we consider the potential applications and the limitations of this new tool.

### Implementation

#### Probability model

**Fig. 1:**
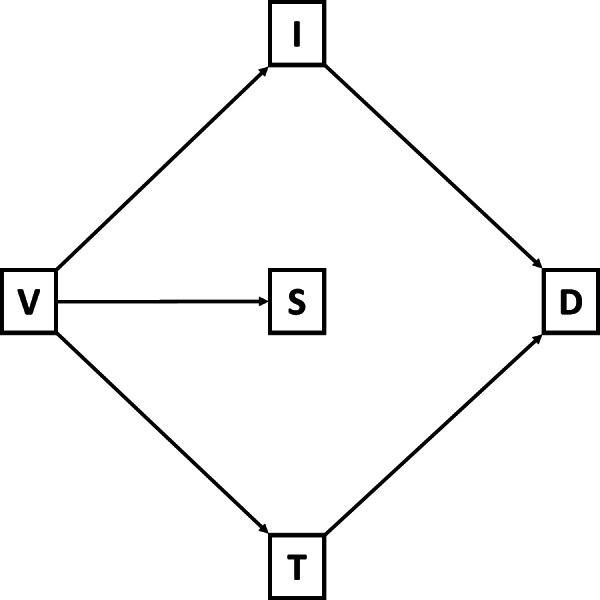
Directed acyclic graph of dependencies in the proposed model. Arrows indicate the direction of causal influences. Note that infection (*I*) can only cause testing (*T*) through the presence of symptoms (*S*), and symptoms can only cause detection through testing. Also, note that vaccination (*V*) is independent of all other variables

The application allows easy usage of an underlying probability model. This model considers five binary random indicator variables: vaccination ($V$), infection ($I$), symptoms ($S$), testing ($T$), and detection ($D$). Each variable indicates the presence or absence of the associated state for an individual at a particular point in time, for example, $V=1$ indicates that an individual is vaccinated, while $T=0$ indicates that an individual has not been tested today. $\mathbf{P}\left(V\right)$, $\mathbf{P}\left(I\right)$, $\mathbf{P}\left(S\right)$, $\mathbf{P}\left(T\right)$, and $\mathbf{P}\left(D\right)$ represent the probability of an individual being in the respective state; equivalently, they can represent the proportion of individuals in an organization expected to be in that state. The resulting model is a joint distribution of these variables, derived analytically from user-customized or default inputs. The general equation for this distribution is Eq. 1, which is represented graphically in Fig. 1. For details of the derivation, see Additional file 1.

*Equation 1: Probability model underlying* ***covidscreen***

$$\mathbf P\left(V,I,S,T,D\right)=\mathbf P\left(V\right)\cdot\mathbf P\left(I\vert V\right)\cdot\mathbf P\left(S\vert I,V\right)\cdot\mathbf P\left(T\vert S,V\right)\cdot\mathbf P\left(D\vert T,I\right)$$

### Backend development

This model is implemented in the *R* programming language (v4.1), primarily using the *data.table* package (v1.14) for data manipulation and *Shiny* framework (v1.7) for communication with the UI [18–20]. The probability model above is implemented using a series of full joint distributions and multiplication operations between each term (distribution) in Eq. 1, starting with the first and proceeding to the right. Probabilities within each term are calculated using the parameters passed from the UI and the equations in Additional file 1.

Inputs are passed from the UI and grouped into categories corresponding to variables in the probability model. After grouping, inputs are validated to ensure proper support. If any validation fails, calculation is halted, and the user is shown a warning until input is corrected. After validation, inputs are converted to corresponding probabilities and used to calculate the conditional distributions of each variable. The full probabilistic model is implemented correspondingly Eq. 1. Details can be found in Additional file 1.

**Fig. 2:**
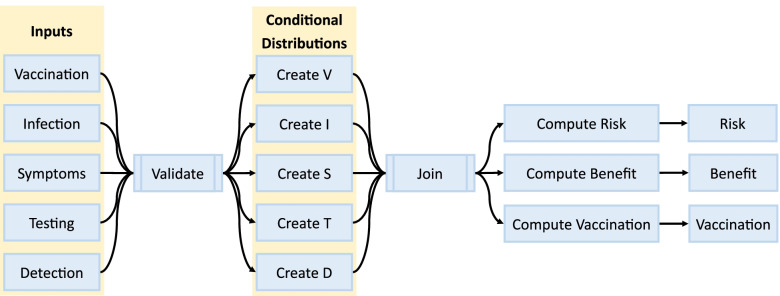
Backend Computation Graph. Inputs are gathered and validated prior to use. If validation fails, no further computation occurs. Conditional distributions of each variable are created using these inputs, then joined and multiplied sequentially to build up an unconditional distribution. This distribution is summarized for each output, and results are passed back to the user interface. This process occurs after each change to user input, but only the necessary components are updated each time

The outputs available in the UI are calculated by filtering to specified variable values and summing over corresponding probabilities. For example, the probability of “undetected cases” is the sum of all probabilities where I = 1 and D = 0. *Shiny*’s reactive programming framework allows outputs to update in real time as users change their inputs. A simplified version of the reactive graph (i.e., the backend architecture) is shown in Fig. 2.

The *R* package is freely available to *R* users. Summary functions are provided for the risk-based metrics in the web application; a suite of classification metrics for the asymptomatic testing program is also provided to aid more advanced performance assessments.

### Frontend development

The UI is implemented in *R Shiny* [18, 20]. Interactive graphics are displayed using an *R* interface to the Highcharts library [21, 22]. The interface is organized as a website, with a landing page, a “Scenarios” tab, a “Profiling” tab, and an “FAQ” tab. External links to code, paper, and documentation are included as well.

Primary outputs are presented on the “Scenarios” tab in graphical and text format; more detailed outputs can be created in the “Profiling” tab. The presented outcomes were chosen based on the authors’ experiences in evaluating asymptomatic testing strategies for an organization throughout the pandemic. The text and graphical representations were chosen through consultation of best practices for health risk communication [23].

### Inputs

Inputs in the UI are grouped into three categories: “Organization”, “Community”, and “Advanced”; by default, “Advanced” inputs are hidden and only available when users invoke it. Probabilistic inputs are shown as sliders between 0% and 100%; numeric inputs are displayed as numeric boxes. A full list of user inputs and their default values is shown in Table 1.

Organizational input, including the frequency of asymptomatic testing in vaccinated members, frequency of testing unvaccinated members, proportion of the organization vaccinated, and size of the organization are listed in this section. Community inputs are the number of daily cases per 100,000 people and the proportion of the community vaccinated.

Advanced parameters are split into three sub-sections: Vaccine Efficacy, Symptoms, and Testing. Vaccine efficacy is the relative reduction in infection risk for vaccinated population compared to the unvaccinated population. Symptom parameters include the time from exposure to symptom onset, the duration of symptoms, if an asymptomatically infected person eventually becomes symptomatic, the proportion of vaccinated cases that eventually show symptoms, and the proportion of unvaccinated cases that eventually show symptoms. Testing parameters are the likelihood of a symptomatic individual seeking testing (assuming timely symptomatic testing and isolation options are readily available), test sensitivity, and test specificity.

**Table 1:** User inputs and defaults

**Organization**		
*Input*		*Default*
Organization Size		1000 people
Vaccinated Testing Frequency		0^a^ days
Unvaccinated Testing Frequency		7 days
% Organization Vaccinated		50%
**Community**		
*Input*		*Default*
Daily Cases per 100k		250 new cases
% Community Vaccinated		50%
**Advanced**		
*Group*	*Input*	*Default*
V	Vaccine Efficacy	30%
T	Test Sensitivity	85%
T	Test Specificity	100%
T	% Symptomatic Tested	100%
S	Symptomatic Period	5 days
S	Pre-symptomatic Period	5 days
S	% Symptomatic: Unvaccinated Cases	50%
S	% Symptomatic: Vaccinated Cases	15%
S	% Symptomatic: Non-Cases	2%

Advanced parameters are split into groups for vaccination (V), testing (T), and symptoms (S), and are intended to need minimal user input. Organization and community parameters are intended to be set by users; the defaults are set to values that provide illustrative outcome plots to assist user comprehension. Note: ^a^ 0 indicates no screening

### Scenarios

The “Scenarios” tab contains three graphics and accompanying text. The first graphic displays on-campus infection risk represented by undetected infections using a dot plot with one dot for each individual in an organization. The expected number of undetected cases is colored red; cases detected due to regular asymptomatic testing are blue, cases identified from symptom-based testing assumed available on-campus are blue gray, and healthy individuals are light gray.

The second graphic represents risk reduction using a stacked bar chart, with cases detected from regular asymptomatic testing represented by blue area and undetected by red. The stacked total is the baseline number of undetected cases with no asymptomatic testing.

The last graphic compares the cases detected per 100 tests in asymptomatic vaccinated and unvaccinated individuals. This is shown as a two-column chart; the accompanying text displays the relative benefits of testing in the higher-risk category, usually the unvaccinated group.

### Profiling

The “Profiling” tab presents the same metrics and inputs as the scenarios but allows users to customize a chosen condition across a range of values and observe the effects on each metric. Graphical outputs are simplified to highlight changes across values. Healthy individuals are not included in the absolute risk graphic, which is displayed as a stacked area chart. Relative risk reduction is displayed as a percentage of total risk, standardizing comparison of effects when total case rates change. Cases detected per 100 tests are displayed as lines, rather than bars.

## Results

### Design and usage

**Fig. 3:**
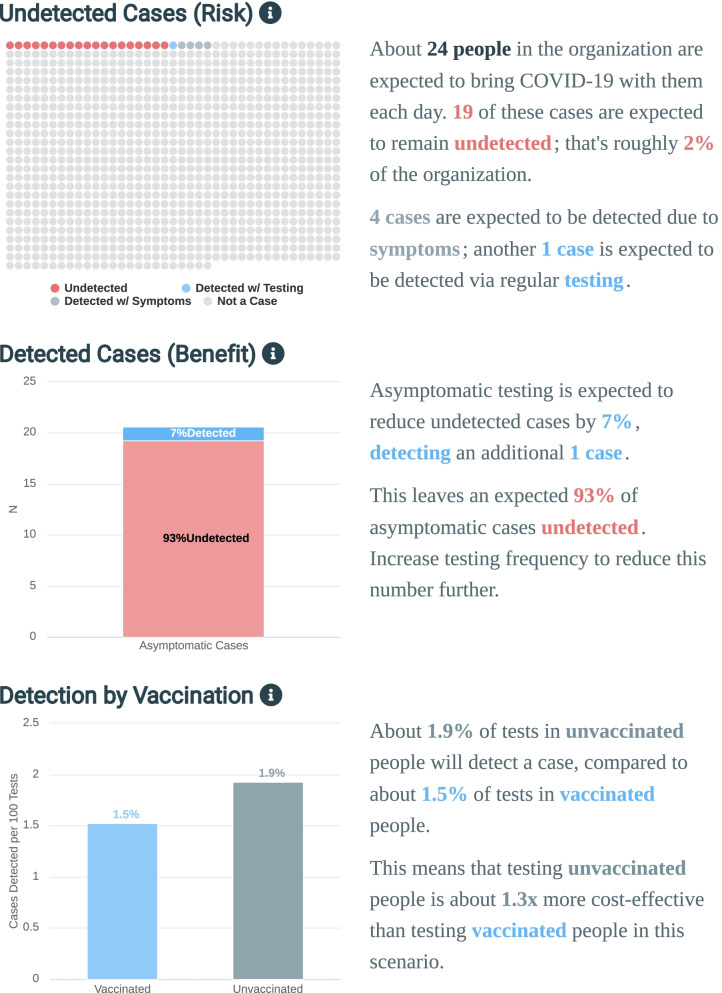
Scenarios UI. Users are guided through risk, benefit, and directions for next steps in the three graphical outputs and accompanying text. The dot plot at the top represents absolute risk as undetected cases. The stacked bar chart in the middle represents benefit as risk reduction/detected cases. The bar chart at bottom represents effectiveness of testing by vaccination status as percentage of positive tests in each group. Inputs are shown at right

Users are presented with similar interfaces in both main tabs. Outputs are positioned centrally, with inputs added to the right (in Scenarios) or bottom (in Profiling). Representation of risk as a dot plot is often associated with more accurate assessments of risk (3). Exact proportions can be shown by hovering over a group. Risk reduction is visually represented as a proportion of the baseline; the y-axis provides absolute numbers, while labels provide relative proportions. Detected cases by vaccination status are shown side-by-side and provide values by group as well as a multiplicative factor for the effectiveness of testing in the group with higher detection rates. Together, these present the user with a top-down narrative highlighting the assessment of interest (undetected cases), benefits of the proposed solution (risk reduction), and directions for further inquiry (vaccination group with the highest benefit gained from additional testing). See Fig. 3 for an example of the Scenarios UI.

Profiling is presented somewhat differently than individual scenarios. Users are only shown one graphic at a time to minimize cognitive overhead associated with filtering multiple sources of information. The input to profile can be chosen through a dropdown menu; the chosen input converts from a one-input element to a two-input element. The output of interest can be selected through a horizontal bar with three output options. In addition, users are given a calculate button that must be clicked to update the graphics; this prevents unnecessary profile calculations, which can increase latency.

This combination of single-scenario summaries and effect profiles allows users to quickly evaluate proposed asymptomatic testing strategies for a given context and further explore the ramifications of changing the assumptions underlying that evaluation. The utility of this approach can be seen in a real-world asymptomatic testing program assessment.

### Case study

St. Jude Children’s Research Hospital (SJCRH) is a pediatric hospital focused on advancing the cure and prevention of catastrophic pediatric diseases through research and treatment with approximately 5,000 employees. Most patients are either very ill, immunocompromised, or both, and thus the acceptable on-campus risk of COVID-19 transmission is therefore very low. SJRCH initiated an asymptomatic employee COVID-19 testing program in March 2020, along with other on-campus non-pharmaceutical intervention measures to minimize the on-campus risk, including employee and patient exposure. This program was re-evaluated on several occasions as the pandemic changed using results from the model presented here. Three of these evaluations are mocked to present a case study of the usage and real-world utility of covidscreen; the parameters in these case studies are shown in Table 2.

**Table 2:** SJCRH parameters

Parameter	Original + No Vaccination	Alpha + Vaccination	Omicron + Vaccination
Organization Size	5000 people	5000 people	5000 people
**Unvaccinated Testing Frequency**	**0** ^**a**^**/3/7/14/30 days**	**0** ^**a**^ **/7 days**	**0** ^**a**^ **/7 days**
**Vaccinated Testing Frequency**	***N/A***	**0** ^**a**^ **/7 days**	**0** ^**a**^ **/7 days**
**% Organization Vaccinated**	**0%**	**0/25/50/75/100%**	**80%**
**Daily Cases per 100k**	**50–500 per 100k**	**50/100 per 100k**	**500 per 100k**
**% Community Vaccinated**	**0%**	**30%**	**60%**
**Vaccine Efficacy**	***N/A***	**90%**	**20/40/60/80%**
Test Sensitivity	95%	95%	95%
Test Specificity	100%	100%	100%
% Symptomatic Tested	100%	100%	100%
Symptomatic Period	5 days	5 days	5 days
Pre-Symptomatic Period	5 days	5 days	5 days
% Symptomatic: Unvaccinated Cases	50%	50%	50%
% Symptomatic: Vaccinated Cases	50%	50%	50%
% Symptomatic: Non-Cases	0%	0%	0%

The SJCRH testing program is evaluated in three sequential scenarios: original variant w/ no vaccination, alpha variant w/ partial vaccination, and omicron variant w/ higher vaccination. Parameters that vary among scenarios are in bold. Some constant parameters differ from the application defaults to match the SJRCH context and settings used in the original evaluations. ^a^ 0 indicates no screening

### No vaccination

**Fig. 4:**
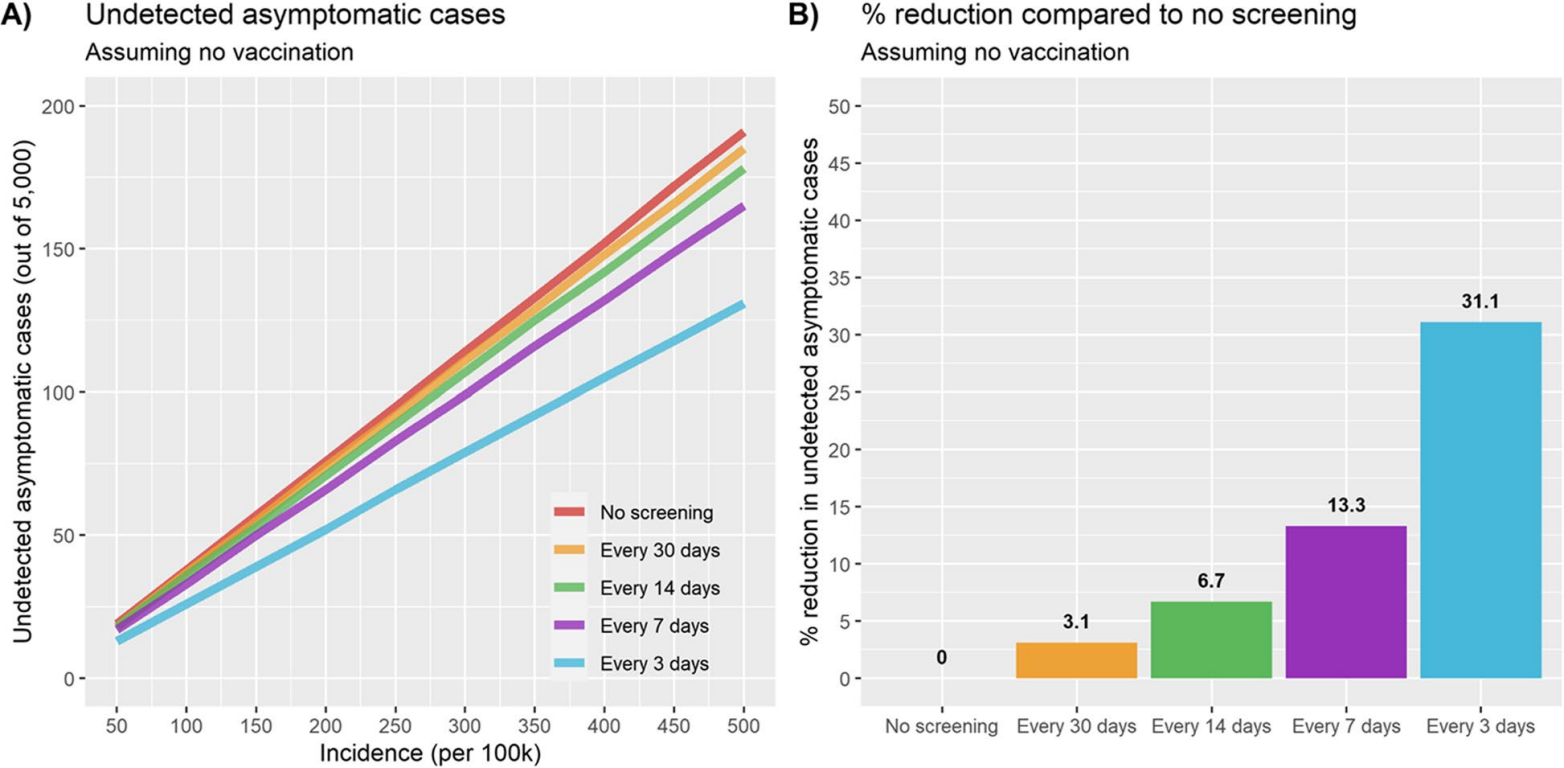
Undetected asymptomatic cases and their reduction due to screening across testing frequencies, assuming no vaccination. **A** shows undetected asymptomatic cases by test community incidence and organization testing frequency; **B** shows the percentage of asymptomatic cases detected via screening by test frequency. Number of undetected cases is shown for a range of incidence rates; percentages are constant across incidence rates and increase more quickly as test frequency increases. Default values were used for other input parameters

In March 2020, SJRCH was faced with the need to maintain operations after much of the world faced quarantine restrictions in the opening phase of the COVID-19 pandemic. To minimize the risk of on-campus transmission, guidelines such as mask-wearing, physical distancing, and minimizing contact between clinical and non-clinical areas were instituted. A COVID-19 asymptomatic testing program is significantly more expensive than these measures, however, and the benefits of such a program were unclear. The primary modeling focus was whether testing (including asymptomatic employee PCR-based testing) could provide a meaningful level of risk reduction, and at what community risk level asymptomatic testing would be needed to bring on-campus risk down to an acceptable level. Using the parameters in the first scenario of Table 2, asymptomatic testing was found to reduce the number of on-campus undetected cases by 3.1% with a 30-day asymptomatic testing frequency, 6.7% with a 14-day frequency, 13.3% with a 7-day frequency, and 31.1% with a 3-day testing frequency. These results can be seen in Fig. 4, along with the number of undetected cases expected across a range of case rates, assuming symptomatic individuals were quickly picked up via testing and isolation without delay. These testing strategies would require an average of 1,167, 2,500, 5,000, and 11,667 tests per week. Risk reduction is shown proportional to the number of tests performed using the proposed model.

### Vaccination rate w/ vaccines having high efficacy

**Fig. 5:**
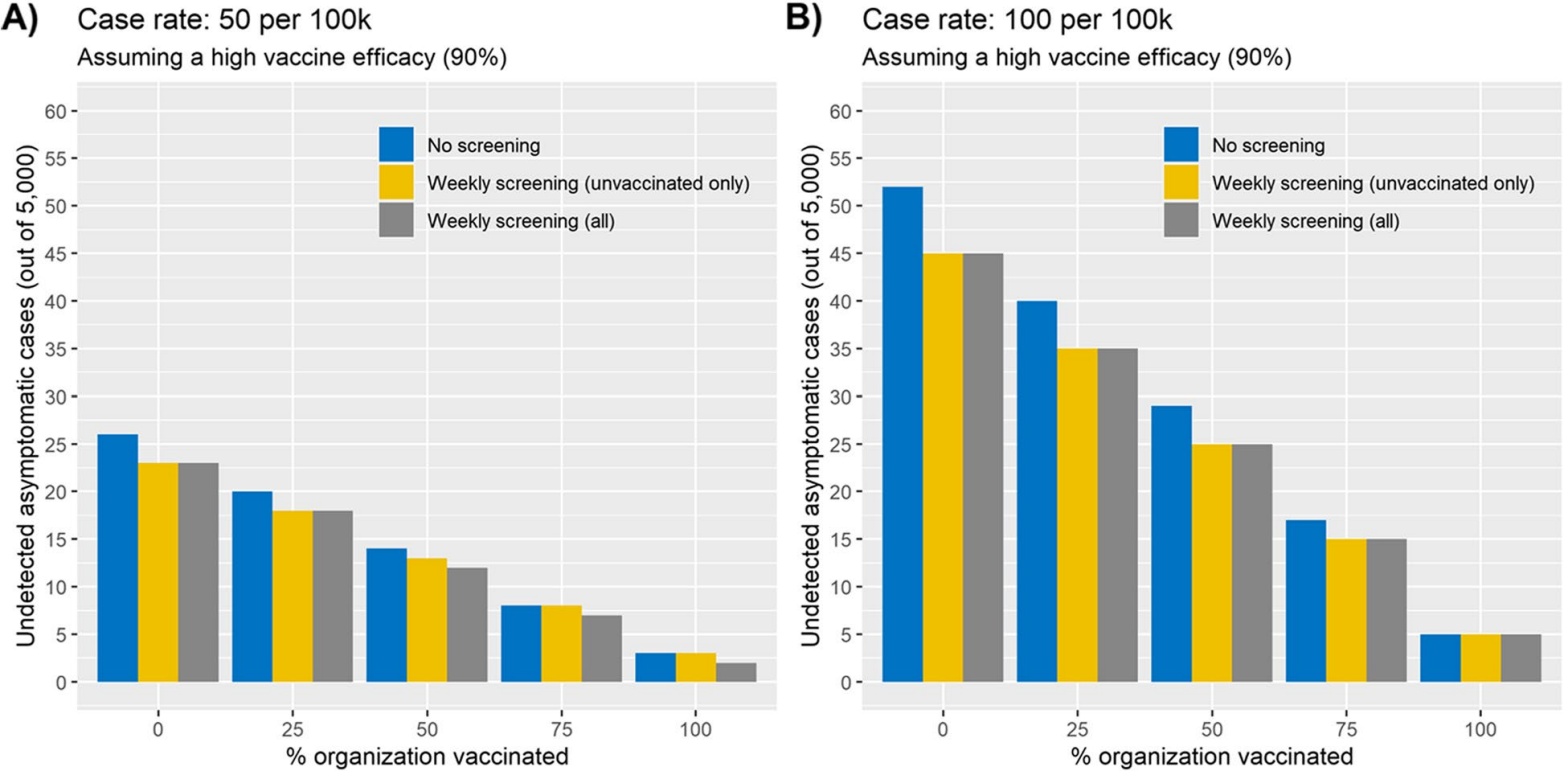
Undetected asymptomatic cases by organization vaccination rates across testing frequencies, assuming 90% vaccine efficacy. **A** shows undetected asymptomatic cases under low case rates; **B** shows the same under moderate case rates. Higher vaccination rates are linearly related to lower number of undetected cases. Weekly (7 day) unvaccinated testing alone is equal to weekly testing across the entire organization. Default values were used for other input parameters

Roughly one year later, COVID-19 cases had declined consistently for some time in the SJCRH region, even as the Alpha variant became dominant. SJCRH vaccine uptake was high, but all employees were still required to undergo regular, asymptomatic testing. The focus of modeling shifted to determine whether testing needed to be maintained at the same rate for vaccinated versus for unvaccinated employees, and to estimate the vaccination rate at which the testing strategy should be reconsidered. Simulation results showed that undetected cases decreased rapidly and linearly as the organization vaccination rate increased (Fig. 5). At a daily incidence rate of 100 cases per 100,000 people, a 75% organization vaccination rate could reduce the number of undetected cases from 45 to 15 without testing vaccinated individuals, which was lower than the number of undetected cases at half that incidence when no vaccination was present (see Fig. 4). Testing restricted to the unvaccinated would require 1,250 tests per week, compared to 11,667 for 3-day testing of all employees.

### Vaccination rate w/ vaccines having varying efficacy

**Fig. 6:**
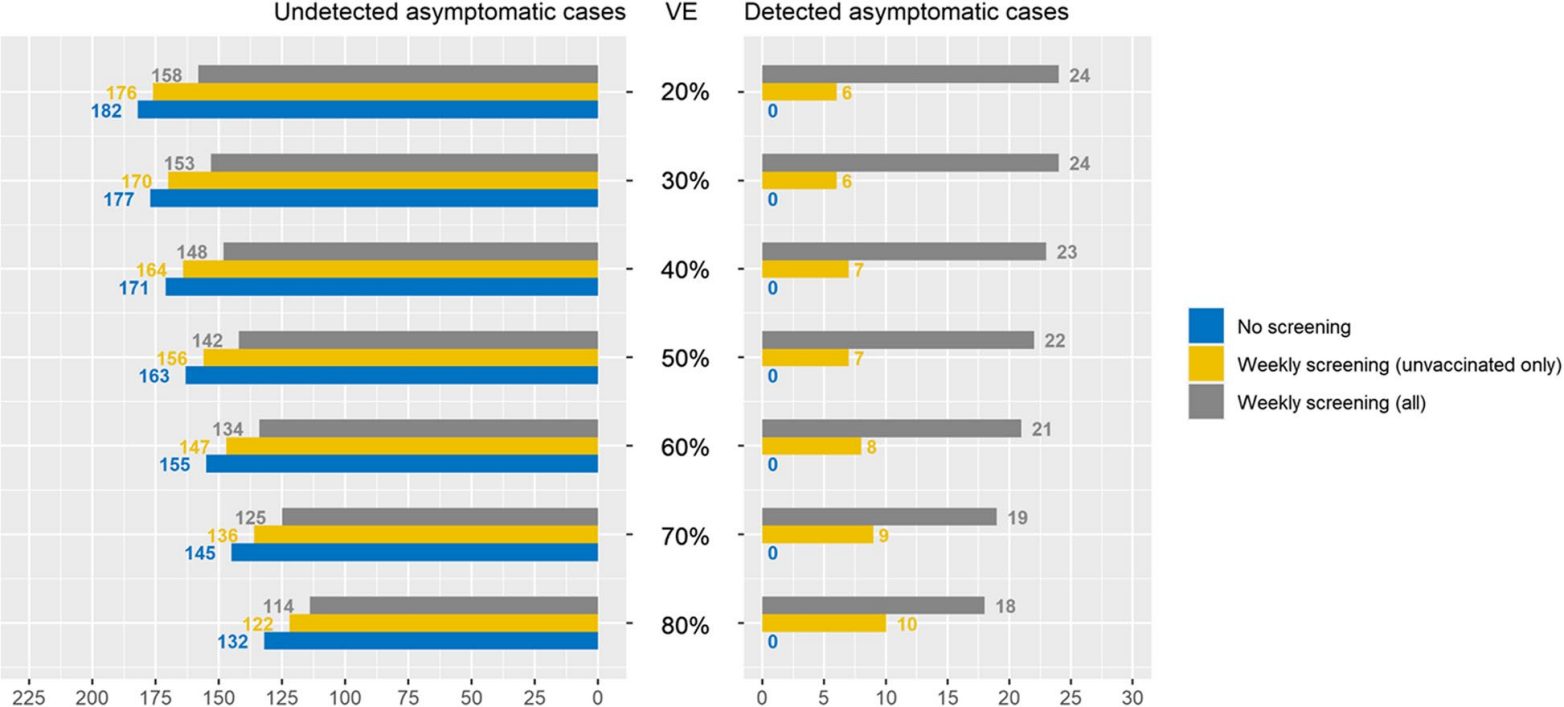
Detected/undetected asymptomatic cases by vaccine efficacy across testing frequencies, assuming high vaccination and case rates. Low vaccine efficacy increases the number of overall cases; weekly screening detects increasing number of vaccinated cases but is insufficient to fully control the resulting risk. Default values were used for other input parameters

At the end of 2021, the Omicron variant first emerged in South Africa and then rapidly overtook the previously dominant Delta variant. Omicron appeared to evade immune response based on exposure to previous variants, rendering natural immunity and vaccination less protective [24]. As incidence rates in the region increased, there was a need to investigate the potential of added value of resumed asymptomatic testing in fully vaccinated employees. Modeling results at transmission rates showed that the number of undetected cases increased as vaccine efficacy decreased in such a highly vaccinated organization. Due to a concurrent higher level of vaccination than in the surrounding community, similarly, the absolute number of unvaccinated cases detected decreased as more vaccinated people were infected, but this was still more than offset by the increased number of vaccinated cases (Fig. 6). Adding asymptomatic testing of vaccinated screening could only offset a 10 – 20% drop in efficacy across a range of possible efficacies. In a population of 5,000 employees, this translates as follows: If vaccine efficacy was originally 80%, undetected cases with weekly unvaccinated asymptomatic screening at an incidence of 500 per 100,000 people would be 122. At 70% effectiveness, testing vaccinated asymptomatic individuals results in 125 undetected cases, and this number increases as vaccine efficacy continues to go down.

## Discussion

Institutional policymakers may find the results above useful but limited. They are situation-specific to an extent but cannot capture every nuance of real life, and they require contextual interpretation for action.

In the “No Vaccination” scenario, the benefits of testing rise with test frequency; however, the number of tests (and the associated costs) also scale with test frequency. An organization might thus adopt a hybrid approach to lower costs; for example, clinical staff might be tested every 3 days, but non-clinical (lower risk) staff may test every 7 days.

Likewise, in the “High Efficacy Vaccines” scenario, a perspective weighing the balance between resources spent on testing and benefit gains suggests a compromise; testing asymptomatic, vaccinated individuals may add very little benefit for the large costs incurred. These results, together with other perceived employee health benefits, could support a push towards high organizational vaccination rates, retaining testing of unvaccinated individuals to further mitigate on-campus risk, which was exactly one of the hypothetical scenarios presented at SJCRH for decision-making. As many healthcare organizations, SJCRH promoted its vaccination program and yielded a highly vaccinated campus.

Further, in the “Varying-Efficacy Vaccines” scenario, asymptomatic testing at acceptable levels simply cannot not compensate for decreased vaccine efficacy with high COVID-19 transmission. Booster shots could be recommended and incentivized as a likely effective path for risk reduction, together with other measures. Note the evolution of disease severity was not accounted for in the model.

It should also be always noted that we are not advising an organization to use asymptomatic testing as a stand-alone strategy. Like many other organizations, our institution also uses a multi-layered intervention. Asymptomatic testing may be made more efficient using strategies such as the group testing procedures mentioned above. Aside from asymptomatic testing, vaccination promotion among employees, patients and patients’ families, masking, social distancing, and zoning the campus have been implemented. A smartphone-based daily symptom screening among employees has also been applied.

### Limitations

Though useful as an efficient and intuitive decision-making tool, covidscreen should be noted as a predictive model and cannot guarantee exact forecasts of future case rates or risk. Model and UI design focus on capturing macro-scale mechanisms and providing actionable, interpretable results. The model evaluates risk in an ideal “steady-state” scenario, where incidence and population at risk are not dynamic for the evaluation period. This assumption should hold safely across short timespans but is expected to break down when applied to highly dynamic scenarios, as well as to extremely high case rates that deplete the population at risk very quickly, although such a scenario seems unlikely. Additionally, given that asymptomatic testing at the community level is usually uncommon, reported public case rates are nearly always an underestimate; in fact, the case rates in the above scenarios are double the reported community rates for that time-period, and a higher multiplier can be possible. As a rule, the model takes user-defined parameters at face value; this provides transparency but also increases the a priori knowledge burden on the user to ensure realistic parameters. This limits the practical use of this tool to settings with a high degree of knowledge and expertise in all aspects of virology, epidemiology, and testing related to an ongoing pandemic. Even in the latter case, results must be viewed in the context of potential departure in those assumptions, because some simplifying assumptions were applied to reduce the number of inputs needed from the user, including assumptions regarding test properties and the length of pre-symptomatic, infectious and symptomatic periods. In particular, the model does not account for individual variability in host characteristics (e.g., vaccine efficacy) or test properties (e.g., positive tests long after a host is no longer transmissible); while this is not expected to adversely affect decisions at the population level, it may lead to poor outcomes for subsets of individuals with characteristics far from the population average. In addition, the tool cannot exhaust all decision-making factors into account, including practical testing capacity, population acceptance of proposed intervention, and potential or likely evolving disease severity, among other issues. Furthermore, formal cost-effectiveness analysis is not the aim of the tool, which may hinder the direct application to the budgetary process, although the tool does offer an assessment of the number of tests required. This is a potential area for future contribution or extension.

## Conclusions

We developed a user-friendly UI, covidscreen, to assess the added value regular, asymptomatic disease testing in an organization, for potential on-campus infectious diseases risk mitigation. The tool offers an intuitive display for an easy comparison of various user-defined scenarios, including various testing frequencies or strategies, which may be informative in decision-making and institutional planning. Formal cost input is not included in the UI, however, by showing the benefit again by comparing different strategies, including testing vaccinated vs. unvaccinated employees and modifying the program when the vaccine efficacy wanes, actionable judgements can be made regarding cost versus potential gain. Despite the limitations discussed above, the tool can be helpful in providing quick, approximate and intuitive output to facilitate early and timely decision-making. Another primary advantage of this tool is that the idea does not only apply to COVID, while it can be readily adapted to epidemic or pandemic arising from other viruses without much foreseen difficulty, offering a potential of wide future usage.

## Availability and requirements

**Project name:** covidscreen

**Project home page:** https://sjbiostat.shinyapps.io/covidscreen/

**Operating system(s):** Platform independent

**Programming language:** R

**Other requirements:** R 4.0 or higher

**License:** GPL-3

**Restrictions to use by non-academics: **None

## Supplementary Information

**Additional file 1.**

## Data Availability

The web application is freely available at https://sjbiostat.shinyapps.io/covidscreen/, and the *R* package by the same name is available at https://jesse-smith.github.io/covidscreen. Source code is available at https://github.com/jesse-smith/covidscreen. Data sharing is not applicable to this article as no datasets were generated or analyzed during the current study.

## Abbreviations

UI: User interface
SJCRH: St. Jude Children’s Research Hospital
