# Supplementary material for: covidscreen: a web app and R Package for assessing asymptomatic COVID-19 testing strategies
Source: BMC Public Health. 2022 Jul 15;22:1361. doi: 10.1186/s12889-022-13718-4 (PMC9284969; doi:10.1186/s12889-022-13718-4)
Supplement: Supplementary file 1 — Additional file 1. [file 12889_2022_13718_MOESM1_ESM.docx]

Additional file 1

# Overview

At its essence, the probabilistic model described below simply splits a population into categories based on a few discrete variables. Reality is significantly more complex, and this model should not be used to provide exact predictions about the future. Instead, it provides a quick tool to help decision-makers construct informed judgments about COVID-19 testing programs, without wading too much through epidemiological and statistical details.

In the sections below, the full model is shown in detail.

# Variables and Parameters

The discussed model is a joint distribution of five binary random variables representing whether an individual is: **Vaccinated** ($V$), **Infected** ($I$), **Symptomatic** ($S$), **Tested** ($T$), or **Detected** ($D$). These variables can take values and jointly form the distribution $P$. Each variable’s expected value is informed directly by a set of parameters and a function of those parameters. Dependencies between two variables are also informed by parameters and a function of those parameters.

While the user interface allows full manipulation of the parameters (assumptions) governing $P$, it makes a distinction between parameters of interest and auxiliary parameters. The parameters of interest are easily manipulated in the user interface (UI); they are either **interventions** that a decision-maker can change, or **context** that varies by physical location. The auxiliary parameters are less visible in the UI; they largely do not vary by location and are typically stationary properties of COVID-19, vaccinations, and tests. Discussion of parameters are ordered below using this distinction.

## Intervention Parameters

The primary intervention of interest is asymptomatic testing (*T*). The probability of an asymptomatic individual being tested on a given day is equal to the frequency of this testing. As organizations may wish to test unvaccinated and vaccinated individuals at different intervals, we distinguish between asymptomatic testing frequency (probability) in the unvaccinated group ($p_{T|S=0,V=0}$) and in the vaccinated group ($p_{T|S=0,V=1}$). The reciprocal of these frequencies is the interval between testing (days); these intervals are assumed to be discrete and are two primary inputs in the user interface.

The additional opportunity for intervention in this model comes from vaccination ($V$). The proportion of the organization vaccinated ($p_{V}$) is also a parameter of interest and a primary input in the UI.

## Contextual Parameters

While the larger community context is not under the direct control of organizational leaders, it is widely variable both across geography and time. We choose to highlight two of these parameters whose values are likely to be available publicly. The first is community incidence rate, or the current rate of new cases per 100,000 in the region. This is equivalent to the probability of becoming infected in the community and is denoted $p_{i_{c}}$. Additionally, we parameterize the proportion of the community vaccinated ($p_{V_{c}}$). Note that this is distinct from the proportion of the *organization* vaccinated, which may or may not equal the vaccinated proportion in the community.

## Auxiliary Parameters

A variety of additional inputs govern $P$. While these may be very uncertain, they are assumed to be largely independent of both context and organizational intervention. These are mostly properties of the pandemic itself, vaccinations, and tests. These parameters remain manipulable in the UI but are hidden by default. Unless a user is confident in customized assumptions, default values are suggested.

### Illness and Symptoms

Two illness-related inputs govern $P$. The first is the pre-symptomatic period ($\tau_{\text{presymp}}$), and the second is the symptomatic period ($\tau_{\text{symp}}$). These are the number of days an infected case is infected but not symptomatic, or infected and symptomatic, respectively.

Some infections may never display symptoms. Since this proportion is likely to vary between vaccinated and unvaccinated infections, it is allowed that the probability of becoming symptomatic to be set separately for unvaccinated ($p_{S|I=1,V=0}$) and vaccinated ($p_{S|I=1, V=1}$) groups. It is further recognized that some individuals will display COVID-like illness (CLI) without being infected. The proportion of *uninfected* individuals is denoted with CLI as $p_{S|I=0}$.

### Vaccination

The primary property of note in vaccination is vaccine efficacy ($e_{V}$). This is the relative decrease in infection risk when comparing vaccinated and unvaccinated group; it is often expressed as a percentage or a probability.

### Testing and Detection

The properties of individual COVID-19 tests determine how accurately they can detect whether someone is or is not infected with COVID-19. This model incorporates two measures of test accuracy. The first is sensitivity ($p_{sens}$), or the proportion of true infections that a test correctly labels as “positive”. The second is specificity ($p_{spec}$), or the proportion of truly uninfected individuals that a test correctly labels as “negative”. It is considered that some symptomatic individuals will not volunteer to be tested, and we denote the probability of being tested if symptomatic as $p_{T|S=1}$. Note that this construct assumes uniform testing accuracy at an institution, which should be broadly approximately true while we acknowledge departure can be expected.

# Probability Model

It has thus far been discussed the variables contained in $P$ and the inputs provided by the user, which can be collectively denoted as $p_{UI}$. $P$ can be formally summarized with respect to these variables as inputs:

$$P=\boldsymbol{P}\left( V=v\cup I=i\cup S=s\cup T=t\cup D=d | p_{UI} \right)$$

To obtain this distribution is a straightforward application of the chain rule, as shown below. Fully expanded, this equation is as follows:

$$\boldsymbol{P=P}\left( V=v\cup I=i\cup S=s\cup T=t\cup D=d | p_{UI} \right)$$

$$P=P\left( V=v | p_{\mathrm{UI}} \right)\cdot P\left( I=i | V=v,p_{\mathrm{UI}} \right)\cdot P\left( S=s | I=i,V=v,p_{\mathrm{UI}} \right)\cdot P\left( T=t | S=s,I=i,V=v,p_{\mathrm{UI}} \right)\cdot P\left( D=d | T=t,S=s,I=i,V=v,p_{\mathrm{UI}} \right)$$

## Vaccination

Beginning with vaccination status, $P\left( V=v|p_{UI} \right)$, the probability of vaccination is a parameter ($p_{V}$):

$$P\left( V=1 \right)=p_{V}$$

$$P\left( V=0 \right)=1-p_{V}$$

## Infection

Next, probability of infection conditional on vaccination is defined as, $P\left( I=i | V=v,p_{UI} \right)$. Incidence in the community ($p_{i_{c}}$), proportion vaccinated ($p_{V}$), and vaccine efficacy ($e_{V}$) are further defined as parameters. It can be shown that the following equations are true:

$$p_{i_{c}|V_{c}=0}=p_{i_{c}}\cdot\frac{1}{1-e_{V}\cdot p_{V_{c}}}$$

$$p_{i_{c}|V_{c}=1}=p_{i_{c}}\cdot\frac{1-e_{V}}{1-e_{V}\cdot p_{V_{c}}}$$

Where the subscript $c$ denotes a community variable or parameter. Since the organization is a sample of the surrounding community, the incidence within the organization is set equal to the incidence outside it.

$$p_{i|V=v}=p_{i_{o}|V_{o}=v_{o}}=p_{i_{c}|V_{c}=v_{c}}$$

Note that, within this model, within organization transmission is ignored and “imported” cases are the focus.

While this offers incidence (the probability of being newly infected), one is interested in the point prevalence (the probability of being currently infected). In the general discrete-time case, incidence can be convolved with the infectious period. However, it is assumed that the infectious period is a constant equal to the pre-symptomatic period added to the symptomatic period, $\tau_{I}$. One may be also interested only in the equilibrium phase of the process, in which the number of added and removed infections are equal. More precisely, one can be concerned with the period after $\tau_{I}$ days of a constant incidence rate. In this phase, the convolution simply becomes multiplication, and it can be shown that

$$\tau_{I}=\tau_{\text{presymp}}+\tau_{\text{symp}}$$

$$p_{I}=\tau_{i}\cdot p_{i}$$

$$\tau_{I}\leq\frac{1}{p_{i}}$$

Where $t_{I}\cdot p_{i}$ must be less than or equal to 1. Note that for infected individuals we assume their pre-symptomatic, symptomatic, and infectious periods do not differ between vaccinated and unvaccinated groups.

Combining the previous 3 results, the following is obtained

$$P\left( I=1 | V=1 \right)=\left( \tau_{\text{presymp}}+\tau_{\text{symp}} \right)\cdot p_{i_{c}}\cdot\frac{1-e_{V}}{1-e_{V}\cdot p_{V_{c}}}$$

$$P\left( I=1 | V=0 \right)=\left( \tau_{\text{presymp}}+\tau_{\text{symp}} \right)\cdot p_{i_{c}}\cdot\frac{1}{1-e_{V}\cdot p_{V_{c}}}$$

$$P\left( I=0 | V=1 \right)=1-\left( \tau_{\text{presymp}}+\tau_{\text{symp}} \right)\cdot p_{i_{c}}\cdot\frac{1-e_{V}}{1-e_{V}\cdot p_{V_{c}}}$$

$$P\left( I=0 | V=0 \right)=1-\left( \tau_{\text{presymp}}+\tau_{\text{symp}} \right)\cdot p_{i_{c}}\cdot\frac{1}{1-e_{V}\cdot p_{V_{c}}}$$

## Symptoms

After obtaining the symptom distribution, $P\left( S=s | I=i,V=v,p_{UI} \right)$. To do so, differential probability of developing symptoms across vaccinated and infected, unvaccinated and infected, and unvaccinated and uninfected groups are accounted for. In addition, the time that eventually symptomatic infections will not display symptoms is accounted for. It is assumed here that vaccination does not affect symptom probability in the uninfected population ($S \perp V|I=0$).

Eventually symptomatic probabilities are given as parameters $p_{S|V=0,I=1}$,$p_{S|V=1,I=1}$, and $p_{S|I=0}$. The probability of an eventually symptomatic infection currently being symptomatic can be obtained by considering the proportion of time they spend symptomatic:

$$p_{S_{\tau}}=\frac{\tau_{\text{symp}}}{\tau_{\text{presymp}}+\tau_{\text{symp}}}$$

The proportion of all infections currently symptomatic is then

$$p_{I_{S}}=p_{S_{\tau}}\cdot p_{S|I=1}$$

And the conditional distributions for symptom status are

$$P\left( S=1 | I=1,V=1 \right)=\frac{\tau_{\text{symp}}}{\tau_{\text{presymp}}+\tau_{\text{symp}}}\cdot p_{S|V=1,I=1}$$

$$P\left( S=1 | I=1,V=0 \right)=\frac{t_{\text{symp}}}{\tau_{\text{presymp}}+\tau_{\text{symp}}}\cdot p_{S|V=0,I=1}$$

$$P\left( S=1 | I=0 \right)=p_{S|I=0}$$

$$P\left( S=0 | I=1,V=1 \right)=1-\frac{\tau_{\text{symp}}}{\tau_{\text{presymp}}+\tau_{\text{symp}}}\cdot p_{S|V=1,I=1}$$

$$P\left( S=0 | I=1,V=0 \right)=1-\frac{\tau_{\text{symp}}}{\tau_{\text{presymp}}+\tau_{\text{symp}}}\cdot p_{S|V=0,I=1}$$

$$P\left( S=0 | I=0 \right)=1-p_{S|I=0}$$

## Testing

For the next term, we consider $P\left( T=t | S=s,I=i,V=v,p_{UI} \right)$. It is assumed that being tested is independent of infection, conditional on symptom and vaccination status ($T \perp I|V,S$); thus, testing depends only on symptoms and vaccination. It is further assumed here that the probability of testing if symptomatic is independent of vaccination status ($T \perp V|S=1$). Parameters the probabilities of testing with symptoms, and without symptoms by vaccination status are given as follows. Assigning conditional probabilities is thus straightforward:

$$P\left( T=1 | S=1 \right)=p_{T|S=1}$$

$$P\left( T=1 | S=0,V=1 \right)=p_{T|S=0,V=1}$$

$$P\left( T=1|S=0,V=0 \right)=p_{T|S=0,T=0}$$

$$P\left( T=0|S=1 \right)=1-p_{T|S=1}$$

$$P\left( T=0 | S=0,V=1 \right)=1-p_{T|S=0,V=1}$$

$$P\left( T=0 | S=0,V=0 \right)=1-p_{T|S=0,V=0}$$

## Detection

The probability of detection, $P\left( D=d | T=t,S=s,I=i,V=v,p_{UI} \right)$, can be derived now. Here, the simplifying but realistic assumption is made that testing will be used to determine someone’s COVID-19 status. That is, regardless of other indicators, an individual will be considered detected if tested positive, and undetected otherwise. Formally, detection to be independent symptom and vaccination status, conditional on testing and infection ($D \perp S,V|T,I$), and detection independent of infection as well when not tested ($D \perp S,I,V|T=0$), are considered. Since $p_{\text{sens}}$ and $p_{\text{spec}}$ are given as parameters, conditional probabilities can be set as follows:

$$P\left( D=1 | T=1,I=1 \right)=p_{\text{sens}}$$

$$P\left( D=1 | T=1,I=0 \right)=1-p_{\text{spec}}$$

$$P\left( D=1 | T=0 \right)=0$$

$$P\left( D=0 | T=1,I=1 \right)=1-p_{\text{sens}}$$

$$P\left( D=0 | T=1,I=0 \right)=p_{\text{spec}}$$

$$P\left( D=0 | T=0 \right)=1$$
